# Heart Failure Readmissions After Cardiac Surgeries: Navigating the High-Risk Terrain∗

**DOI:** 10.1016/j.jacadv.2023.100600

**Published:** 2023-09-08

**Authors:** Waqas Ullah, Indranee Rajapreyar, Yevgeniy Brailovsky

**Affiliations:** Department of Cardiology, Thomas Jefferson University Hospitals, Philadelphia, Pennsylvania, USA

**Keywords:** cardiac surgery, heart failure readmissions

**Figure:**
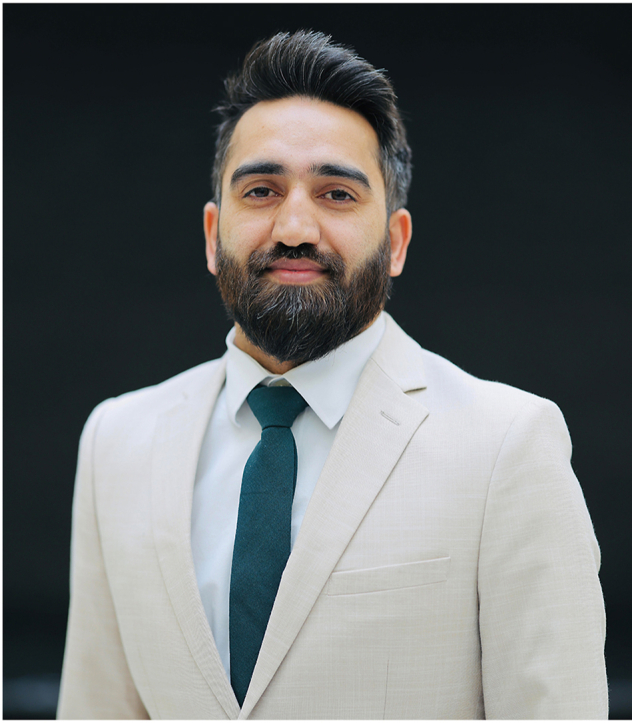

With continuous advancements in cardiac surgical techniques and perioperative management, surgical complications comprise a minority of postoperative readmissions.^1^ An increasing number of readmissions are now attributed to medical complications, principally low cardiac output and heart failure (HF).^1^^,^^2^ As surgeons operate on an ever-aging population with increasingly ambitious procedures, it is imperative to be able to prognosticate adverse outcomes to guide risk stratification and patient selection. Current evidence on the predictors of HF-related readmissions after major cardiac surgeries is sparse and limited to small-scale studies.^2^

The study by Sabe et al identified 9 preoperative and 5 perioperative or postoperative conditions associated with a higher risk of HF-related 30-day readmission after the index cardiac surgical procedure.^3^ Prior history of HF was found to have the strongest association with future HF-related 30-day readmission, increasing the risk by 2.29-fold compared with patients who were not readmitted or admitted for non-HF reasons.^3^ This risk was irrespective of the type of cardiac surgery and other comorbid conditions. This finding aligns with the prior literature that showed that preoperative HF, mainly left ventricular ejection fraction <50%, was an independent variable associated with a greater postoperative risk for HF.^2^^,^^4^, ^5^, ^6^, ^7^, ^8^, ^9^, ^10^

We believe several technical factors and mechanistic explanations exist for this observation. Firstly, it is hard to accurately discern whether the readmissions were due to right ventricular (RV) or left ventricular (LV) failure and if the ejection fraction was reduced or preserved. Additionally, given the limitations of the International Classification of Diseases, it is unknown how the diagnosis of HF was made based on echocardiographic parameters, clinically, or both. In the case of the former, it has long been appreciated that sternotomy and a pericardial incision are obstacles to procuring accurate RV function. The traditionally used tricuspid annular plane systolic excursion and lateral tricuspid annulus peak systolic velocity are inherently reduced postoperatively, have a regional effect, are prone to tethering and translational motion, and thus are not reflective of global RV function in the postsurgical state. Similarly, the right ventricular index of myocardial performance and fractional shortening methods for RV function assessment lack sensitivity and are prone to measurement errors. Mechanistically, RV failure after open heart surgeries is not uncommon due to a high-risk of ischemic insult related to air embolism into the right coronary artery due to its anterior position and high periprocedural volume overload unmasking RV failure.^11^ LV failure after cardiac surgery could be due to metabolic causes (acidosis, hypoxia, hypercapnia), first-time recognition of preprocedural LV dysfunction, periprocedural myocardial infarction, incomplete coronary artery bypass grafting, risk of suturing the left circumflex artery in the atrioventricular groove (due to its proximity to the mitral valve area), and high incidence of postoperative atrial fibrillation (AF) leading to tachycardia mediated cardiomyopathy at 30-day follow-up of index procedure.^10^

Sabe et al also showed that older adults (age >65 years) and female patients had 1.1 to 1.5 times higher odds of HF-related readmission. This could be explained by the accumulation of multiple traditional risk factors for HF in older patients and a higher risk of stress-related cardiomyopathy due to increased catecholamine release during surgery, particularly in females.^12^ Other high-risk factors included diabetes, chronic kidney disease, chronic obstructive pulmonary disease, chronic liver disease, obesity, and AF. Apart from AF, the central mechanism in most conditions is myocardial ischemia leading to HF due to compromised epicardial or microvascular circulation. In the context of cardiac surgery, the risk of HF is further compounded by cardioplegia and prolonged cardiopulmonary bypass-related myocyte apoptosis and reperfusion injury. Such dysfunction could be temporary due to myocardial stunning or persistent due to profound ischemia leading to a 30-day HF-related readmission.^2^^,^^8^ As Redžek et al^2^ showed, longer cardiopulmonary bypass duration reflects the complexity of cardiac surgery and could directly influence late cardiovascular outcomes. For AF, a plausible mechanism for postoperative HF is uncontrolled heart rates and rapid ventricular response leading to diminished ventricular filling time and reduced cardiac output. Studies by Gorter et al and Greenberg et al showed that postcardiac surgery, heart function was more depressed in patients with AF and those with sinus rhythm but a history of prior AF compared with those with no AF.^13^^,^^14^

The perioperative and postoperative factors associated with 30-day HF readmissions included acute kidney injury, temporary mechanical circulatory support device use, and a longer length of stay (≥12 days). It is unclear if these conditions were the consequence or etiology of immediate postoperative HF and subsequent HF readmission at 30 days. Nonetheless, their strong association with HF readmission calls for closely integrated postdischarge management of patients with these features. Lastly, the study showed that nonelective cardiac surgery and nonroutine discharge were also associated with a significantly higher risk of HF-related readmission. This is not unexpected, as it corresponds to providers not having the opportunity to medically optimize the patient either preprocedure (in the case of nonelective surgery) or postprocedure (in the case of nonstandard discharge).

As acknowledged by the authors, the inherent limitations of the Nationwide Readmissions Database and lack of granular data on the specifics of myocardial dysfunction precluded their ability to perform a more robust and stratified analysis. Future prospective studies are needed to validate these findings. A more robust study might seek to compare these high-risk variables in a binary fashion (eg, presence of diabetes vs no diabetes, and so on) to delineate causation with the outcome of interest. Additionally, one way to determine whether these risk factors were valid predictors for HF readmission or merely a temporal association would be to develop training, validation, and testing models by performing c-statistics.

In summary, it is well known that unplanned nonelective HF-related hospital readmissions are associated with increased resource utilization, higher health care costs, and a dismal influence on patient quality of life. Having identified the high-risk pre-, peri-, and post-procedure variables, the study by Sabe et al enables the implementation of rigorous preoperative risk stratification, optimization of high-risk conditions, and management of postoperative complications to reduce readmissions.

## Funding support and author disclosures

The authors have reported that they have no relationships relevant to the contents of this paper to disclose.

## References

[1] Lomivorotov V.V., Efremov S.M., Kirov M.Y., Fominskiy E.V., Karaskov A.M. (2017). Low-cardiac-output syndrome after cardiac surgery. J Cardiothorac Vasc Anesth.

[2] Redžek A., Mironicki M., Gvozdenović A. (2015). Predictors for hospital readmission after cardiac surgery. J Card Surg.

[3] Sabe S.A., Sabe M.A., Kennedy K.F., Sellke F.W., Ehsan A. (2023). Risk factors for heart failure readmission after cardiac surgery. JACC: Adv.

[4] Maganti M.D., Rao V., Borger M.A., Ivanov J., David T.E. (2005). Predictors of low cardiac output syndrome after isolated aortic valve surgery. Circulation.

[5] Maganti M., Badiwala M., Sheikh A. (2010). Predictors of low cardiac output syndrome after isolated mitral valve surgery. J Thorac Cardiovasc Surg.

[6] Mebazaa A., Pitsis A.A., Rudiger A. (2010). Clinical review: practical recommendations on the management of perioperative heart failure in cardiac surgery. Crit Care.

[7] Hannan E.L., Zhong Y., Lahey S.J. (2011). 30-day readmission after coronary artery bypass graft surgery in New York State. J Am Coll Cardiol Intv.

[8] Wallmann R., Llorca J., Gómez-Acebo I., Ortega Á.C., Roldan F.R., Dierssen-Sotos T. (2013). Prediction of 30-day cardiac-related-emergency-readmissions using simple administrative hospital data. Int J Cardiol.

[9] Algarni K.D., Maganti M., Yau T.M. (2011). Predictors of low cardiac output syndrome after isolated coronary artery bypass surgery: trends over 20 years. Ann Thorac Surg.

[10] Ellenberger C., Sologashvili T., Cikirikcioglu M. (2017). Risk factors of postcardiotomy ventricular dysfunction in moderate-to-high risk patients undergoing open-heart surgery. Ann Card Anaesth.

[11] Levy D., Laghlam D., Estagnasie P., Brusset A., Squara P., Nguyen L.S. (2021). Postoperative right ventricular failure after cardiac surgery: a cohort study. Front Cardiovasc Med.

[12] Oh T.K., Song I.A., Park Y.M. (2017). Prevalence and risk factors for postoperative stress-related cardiomyopathy in adults. PLoS One.

[13] Gorter T.M., van Melle J.P., Rienstra M. (2018). Right heart dysfunction in heart failure with preserved ejection fraction: the impact of atrial fibrillation. J Card Fail.

[14] Greenberg J.W., Lancaster T.S., Schuessler R.B., Melby S.J. (2017). Postoperative atrial fibrillation following cardiac surgery: a persistent complication. Eur J Cardio Thorac Surg.

